# Optimization of Friction Stir Welding Tool Advance Speed via Monte-Carlo Simulation of the Friction Stir Welding Process

**DOI:** 10.3390/ma7053435

**Published:** 2014-04-30

**Authors:** Kirk A. Fraser, Lyne St-Georges, Laszlo I. Kiss

**Affiliations:** Research Group for Process and Systems Engineering (GRIPS), Université du Québec à Chicoutimi (UQAC), 555 boul. Université, Chicoutimi, G7H 2B1 Québec, Canada; E-Mails: kirk.fraser1@uqac.ca (K.A.F.); laszlo_kiss@uqac.ca (L.I.K.)

**Keywords:** friction stir welding, tool speed optimization, non-linear, finite difference method

## Abstract

Recognition of the friction stir welding process is growing in the aeronautical and aero-space industries. To make the process more available to the structural fabrication industry (buildings and bridges), being able to model the process to determine the highest speed of advance possible that will not cause unwanted welding defects is desirable. A numerical solution to the transient two-dimensional heat diffusion equation for the friction stir welding process is presented. A non-linear heat generation term based on an arbitrary piecewise linear model of friction as a function of temperature is used. The solution is used to solve for the temperature distribution in the Al 6061-T6 work pieces. The finite difference solution of the non-linear problem is used to perform a Monte-Carlo simulation (MCS). A polynomial response surface (maximum welding temperature as a function of advancing and rotational speed) is constructed from the MCS results. The response surface is used to determine the optimum tool speed of advance and rotational speed. The exterior penalty method is used to find the highest speed of advance and the associated rotational speed of the tool for the FSW process considered. We show that good agreement with experimental optimization work is possible with this simplified model. Using our approach an optimal weld pitch of 0.52 mm/rev is obtained for 3.18 mm thick AA6061-T6 plate. Our method provides an estimate of the optimal welding parameters in less than 30 min of calculation time.

## 1. Introduction

**Figure 1.**
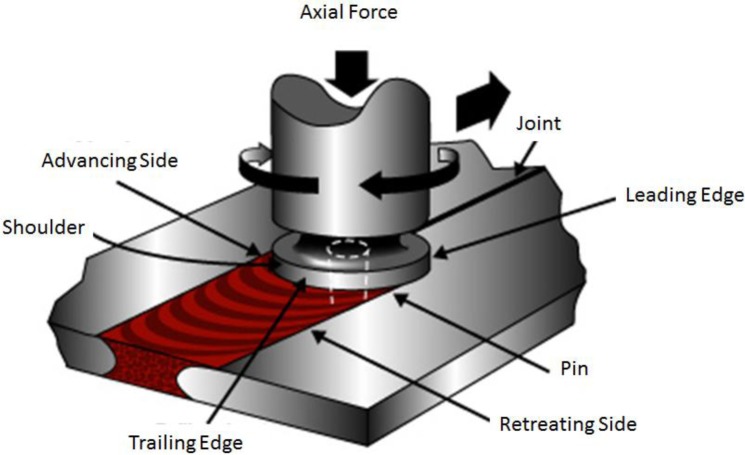
Friction stir welding diagram.

Joining of aluminum by conventional welding methods is problematic at the best of times; defects are common, as is poor post welding strength. Friction stir welding (FSW), a solid-state welding procedure allows joining of aluminum plates with fewer defects and with a very good post welding strength. An image of a FSW apparatus is shown in Figure 1.

The ultimate strength and fatigue resistance of a welded aluminum joint is of critical importance in industries such as aeronautical and aero-space. In recent years, many authors have focused on optimizing the ultimate strength of a friction stir welded joint. Elatharasan and Kumar [1] use the response surface method (RSM) to optimize the strength of the weld of dissimilar 6061-T6 and 7975-T6 aluminum butt joint welds. They show that there exists a range of acceptable rotational and advancing speeds to create a high strength defect free joint.

Balasubramanian and Laksihminarayaranan [2] use the Taguchi method to determine the most important process parameters for a high strength welded joint. They investigate the RDE-40 aluminum material in their study. Their method shows that the advancing speed, rotational speed, and the axial force on the tool are the most important process parameters. They found that the optimum tensile strength obtainable for the RDE-40 material is ~303 MPa. Their results were verified experimentally.

Zhang and Lui [3] investigate optimization of the underwater FSW (UWFSW) process. The UWFSW is a method that involves performing the FS weld in a closed circulation welding chamber filled with water. The method allows for very high strength welds. They experimentally determined a set of data points at various welding speeds, and then fit a response surface with the least squares method. They use the constructed response surface to optimize the weld strength. They show that the tensile strength of the welded joint can be increased by 6% with the UWFSW method as compared to the conventional FSW process. A maximum tensile strength of 360 MPa can be obtained with an advance speed of 223 mm/min and a rotational speed of 983 rpm.

Heidarzadeh *et al.* [4] developed a four parameter model for the tensile strength of pure copper butt welds. They show that the tensile strength of the weld is optimized with an advance speed of 84mm/min and a rotational speed of 942 rpm. The RSM method is used to fit a least squares regression model to the experimentally determined data points.

On the other hand, there has been relatively little research into the optimization of the advance and rotational speed of the FSW tool in order to provide welds that are strong enough (not necessarily optimal strength) and do not have defects. Maximizing the welding advance speed is of great importance for industries, such as structure and bridge fabrication in order to be profitable. Therefore the work presented in this paper is focused on optimizing the speed of advance as opposed to the weld strength. Constraints are applied to the optimization problem to limit the speed of advance, the maximum welding temperature and the speed of rotation.

Ideally, the fastest speed of advance would be used in order for the business owner to minimize welding costs. If the ratio of speed of advance to speed of rotation is not within an acceptable range, there is a high risk of causing welding defects. Leonard and Lochyer [5] provide a description of a few of the most common defects found in a joint created by the FSW process.

Grujicic *et al.* [6–8] use an Arbitrary Lagrangian-Eulerian (ALE) method to predict the material flow and the resulting microstructure of AA5083 butt welded plates. They show that their numerical approach is able to predict general trends in room temperature strength, residual stress as well as grain size (among other parameters). However, the time required to generate one single point with this method can be on the order of hours or even days. With our approach, 100 data points are generated by the finite difference solver within less than two minutes. One can easily see that an attempt to perform process optimization with a fully coupled ALE approach would be prohibitive.

Recent advances in numerical simulation methods such as fully coupled thermo-mechanical smoothed particle hydrodynamics (SPH) have led to very powerful numerical predictions of weld defects. Pan *et al.* [9], as well as Bohjwani [10], use the SPH method to simulate the FSW process for various welding parameters. They show that the SPH method captures the evolution of the free surface (shows possible welding defects). The work of Pan *et al.* [9] gives detailed grain size, hardness, and residual stress evolution using the SPH method. The work of Fraser *et al.* [11] focuses directly on predicting welding defects numerically using the SPH method. However, these fully coupled methods are not suitable at this time for performing process optimization.

We will show that the microstructural evolution of an aluminum FSW joint can be neglected when performing an initial estimation of the optimal process parameters. The evolution of the microstructure is strongly linked to the temperature history and distribution in the work pieces during the FSW process. For this reason, we propose a simplified thermal model that approximates the mechanical effects and associated microstructure changes by using an analytical model.

Currently, state of the art fully coupled simulation models of FSW using SPH or ALE are prohibitively slow and cannot be used for numerical optimization of the process parameters. The proposed Monte-Carlo finite difference method (FDM) optimization approach provides an excellent estimation of the optimal FSW parameters within less than 30 min. The FDM code is able to generate 100 data points in less than two minutes, and the subsequent optimization calculations can be performed in about 20 min. Experimental optimization can take many days to find the optimal parameters. Fully coupled numerical simulation optimization work is likely to take on the order of weeks or months.

Our numerical tactic to optimize the FSW tool advancing speed involves formulating a finite difference solution for the two-dimensional heat conduction equation. A Monte-Carlo simulation (MCS) is then performed to provide a large number of data points to fit a response surface for the advancing speed. Finally the exterior penalty method (EPM) is used to solve for the optimal advancing and rotational speed.

## 2. Finite Difference Formulation

In order to determine the temperature distribution in the aluminum work pieces, the 2D transient conduction equation will be solved:

$$\frac{\partial^{2}T}{\partial x^{2}}+\frac{\partial^{2}T}{\partial y^{2}}+\frac{\dot{q}(T)}{k_{cond}}=\frac{\rho C_{p}}{k_{cond}}\frac{\partial T}{\partial t}$$ (1)

where *T* is the temperature *x* and *y* are the spatial dimensions, *k_cond_* is the thermal conductivity of the work piece, *C_p_* is the heat capacity, ρ is the density of the material and *t* is time. The heat generation term is a combination of the heat loss and heat generation terms. Heat is lost from the top of the work piece surfaces due to convection with the air ( ${\dot{q}}_{conv}$). This means that the heat exchange in the *x*-*y* plane is merged into the volumetric heat source term in Equation (1). The bottom of the work pieces are supported on a steel structure, heat loss at the bottom surface of the work pieces is due to the aluminum in contact with the steel base ( ${\dot{q}}_{conv}$). As such, the $\dot{q}$ term is composed of the preceding terms:

$$\dot{q}={\dot{q}}_{rad}+{\dot{q}}_{conv}+{\dot{q}}_{cond}+{\dot{q}}_{FSW}$$ (2)

where ${\dot{q}}_{rad}={\bar{h}}_{r}(T_{surr}-T_{s})$, ${\dot{q}}_{conv}=\bar{h}(T_{\infty }-T_{s})$ and ${\dot{q}}_{cond}=(T_{\infty }-T_{s})/\left(\frac{t_{steel}}{k_{steel}}+\frac{1}{\bar{h}}\right)$. ${\bar{h}}_{r}$ is the linearized radiation coefficient, $\bar{h}$ is the convection coefficient, *T_surr_* and *T*_∞_ are taken as the atmospheric air temperatures (20 degrees Celsius) and *T_s_* is the nodal surface temperature.

The heat generation term for the FSW process is formulated based on the work of Durdanovic *et al.* [12]:

$${\dot{q}}_{FSW}=\frac{2\pi }{3A_{node}t_{plate}N_{tool}}\omega \mu_{k}(T)pr_{2}{}^{3}$$ (3)

**Figure 2.**
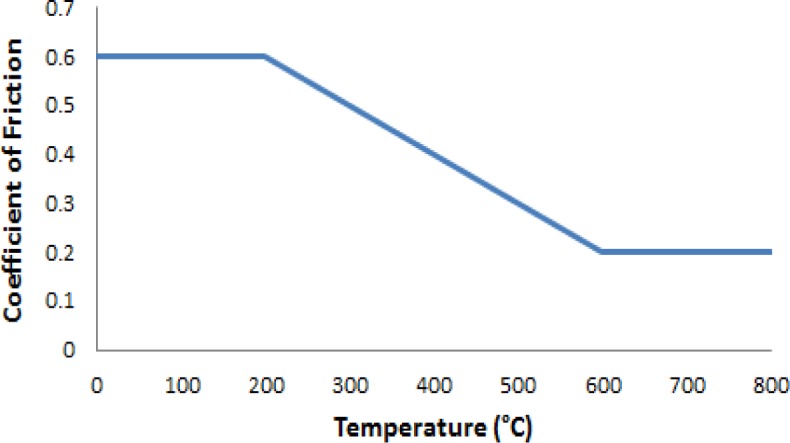
Friction as a function of temperature.

Here we consider a coefficient of friction that is a function of the current nodal temperature (μ*_k_*(*T*), see Figure 2). *A_node_* is the nodal area of the plate, *t_plate_* is the thickness of the plate, *N_tool_* is the number of nodes used to represent the diameter of the welding tool, ω is the angular velocity of the tool, *p* is the axial pressure on the tool and *r*_2_ is the radius of the tool shoulder.

Typically a constant friction coefficient model (Coulomb) is used to evaluate the temperature distribution in the work pieces during the FSW process. The coefficient of friction in reality is a function of many parameters: pressure, speed, temperature, time, *etc.* A stick-slip friction behavior is proposed by various authors including Schneider *et al.* [13]. Including the variable friction function renders the problem non-linear due to the heat generation term.

The solution of the partial differential equation (PDE) will be approximated using the finite difference method. The temporal and spatial derivatives are given by:

$${\frac{\partial T}{\partial t}]}_{i,j}=\frac{T_{i,j}{}^{k+1}-T_{i,j}{}^{k}}{\Delta t}$$ (4)

$${\frac{\partial^{2}T}{\partial x^{2}}]}_{i,j}=\frac{T_{i+1,j}{}^{k}+T_{i-1,j}{}^{k}-2T_{i,j}{}^{k}}{\Delta x^{2}}$$ (5)

$${\frac{\partial^{2}T}{\partial y^{2}}]}_{i,j}=\frac{T_{i,j+1}{}^{k}+T_{i,j-1}{}^{k}-2T_{i,j}{}^{k}}{\Delta y^{2}}$$ (6)

These approximations will be used to formulate the full set of finite difference equations for the FSW process.

More information about numerical solutions of parabolic partial differential equations is available in Farlow [14] among many other references.

**Figure 3.**
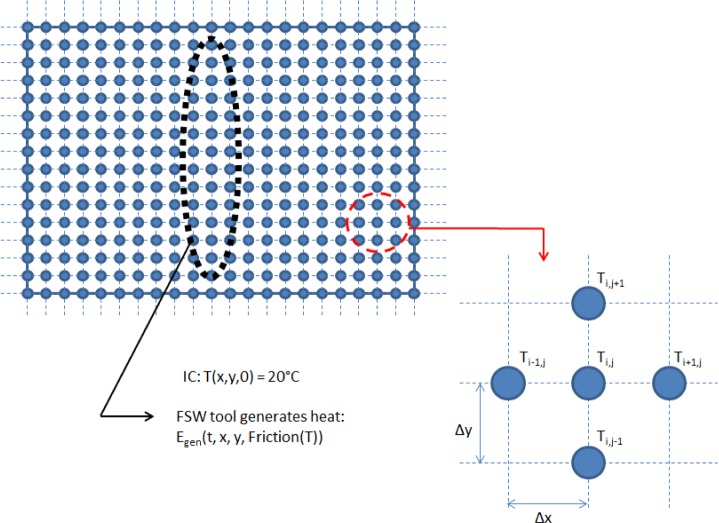
Finite difference grid.

The five point FDM stencil used for the interior nodes is shown in Figure 3. *i* and are *j* the indices for the *x* and *y* spatial directions respectively, *k* is the temporal index and δ_s_ is the nodal spacing (we consider a uniform grid spacing with equal spacing in the *x* and *y* directions). The finite difference formulation for an internal node is:

$$T_{i,j}{}^{k+1}=\frac{k_{cond}\Delta t}{\rho C_{p}\delta_{s}{}^{2}}\left(T_{i+1,j}{}^{k}+T_{i-1,j}{}^{k}+T_{i,j+1}{}^{k}+T_{i,j-1}{}^{k}+\frac{\delta_{s}{}^{2}}{k_{cond}}\dot{q}(T_{i,j}{}^{k})\right)+\left(1-\frac{4k_{cond}\Delta t}{\rho C_{p}\delta_{s}{}^{2}}\right)T_{i,j}{}^{k}$$ (7)

**Figure 4.**
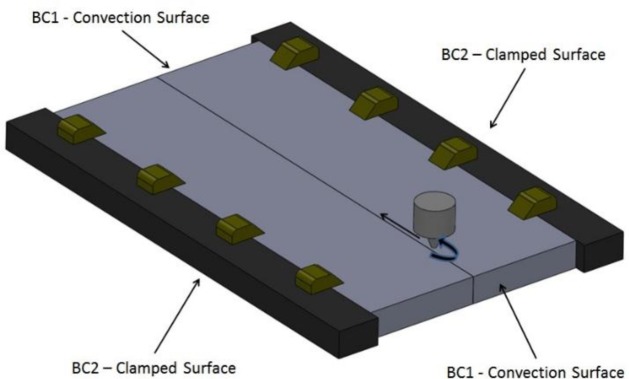
Schematic of FSW setup.

The nodes on the boundary of the work pieces require an alternate formulation due to heat dissipation through convection and radiation. A linearized radiation approximation is used and is combined with the convection term. A schematic of the boundary conditions is shown in Figure 4.

The finite difference formulation for a node on the convection surface boundary is:

$$T_{i,j}{}^{k+1}=\frac{k_{cond}\Delta t}{\rho C_{p}\delta_{\text{s}}{}^{2}}\left(T_{i+1,j}{}^{k}+T_{i-1,j}{}^{k}+2T_{i,j-1}{}^{k}+\frac{\delta_{\text{s}}{}^{2}}{k_{cond}}\dot{q}(T_{i,j}{}^{k})+\frac{2\bar{h}\delta_{s}}{k_{cond}}T_{\infty }\right)+\left(1-\frac{k_{cond}\Delta t}{\rho C_{p}\delta_{\text{s}}{}^{2}}\left(4+\frac{2\bar{h}\delta_{s}}{k_{cond}}\right)\right)T_{i,j}{}^{k}$$ (8)

**Figure 5.**
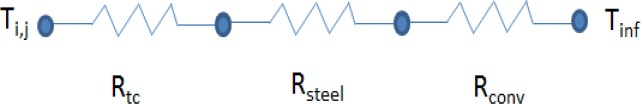
Clamped boundary thermal resistance.

The finite difference formulation for a node on the clamped boundary is:

$$T_{i,j}{}^{k+1}=\frac{k_{cond}\Delta t}{\rho C_{p}\delta_{s}{}^{2}}\left(2T_{i-1,j}{}^{k}+T_{i,j-1}{}^{k}+T_{i,j+1}{}^{k}+\frac{\delta_{s}{}^{2}}{k_{cond}}\dot{q}(T_{i,j}{}^{k})+\frac{2\delta_{s}}{k_{cond}R^{\prime \prime }}T_{\infty }\right)+\left(1-\frac{k_{cond}\Delta t}{\rho C_{p}\delta_{s}{}^{2}}\left(4+\frac{2\delta_{s}}{k_{cond}R^{\prime \prime }}\right)\right)T_{i,j}{}^{k}$$ (9)

where the thermal resistance, $R^{\prime \prime }=R^{\prime \prime }{}_{tc}+\frac{t_{steel}}{k_{steel}}+\frac{1}{\bar{h}}$, ${R^{\prime \prime }}_{tc}$ (2.75 m^2^K/W [15]) is the thermal resistance of aluminum with air at the interface, *t_steel_* and *k_steel_* are the thickness and the thermal conductivity of the steel support. A schematic of the thermal resistance at the clamped boundary is shown in Figure 5.

As the temporal dimension is approximated using the forward difference scheme (explicit integration), the solution is subject to limitation on the time step size to allow for numerical stability of the FDM formulation. More information on the stability of parabolic PDE’s can be found from Morton and Mayers [16]. Typically the boundary nodes will provide the limiting case for the time step.

$$\Delta t=0.9\left[\min\left(\frac{\rho C_{p}\delta_{s}{}^{2}}{4k_{cond}},\frac{\rho C_{p}\delta_{s}{}^{2}}{2k_{cond}\left(2+\frac{\bar{h}\delta_{s}}{k_{cond}}\right)},\frac{\rho C_{p}\delta_{s}{}^{2}}{2k_{cond}\left(2+\frac{\delta_{s}}{kR^{\prime \prime }}\right)},\frac{\rho C_{p}\delta_{s}{}^{2}}{2k\left(2+\frac{\bar{h}{\text{δ}}_{s}}{k_{cond}}+\frac{\delta_{s}}{k_{cond}R^{\prime \prime }}\right)}\right)\right]$$ (10)

A listing of the parameters used in the FDM simulation of the FSW process is shown in Table 1. An initial temperature of the work pieces of 20 degrees Celsius is considered. In this work, we consider a butt weld of two AA6061-T6 plates that are 3.18 mm thick.

## 3. Optimization Approach

The main goal of our work is to optimize the advancing speed of the welding tool during the FSW process. The following steps are undertaken to accomplish this:

(1) Formulate the temperature distribution for the FSW process with a FDM approximation of the transient 2D heat conduction equation;
(2) Perform the MCS by varying the advancing speed and rotational speed of the tool;
(3) Construct a response surface from the MCS data points;
(4) Use the least squares method to fit an analytical surface to the MCS data;
(5) From the LSM, determine the mathematical model for the advancing speed as a function of maximum welding temperature and rotational speed;
(6) Formulate the constraints on the welding parameters;
(7) Use the exterior penalty method to optimize the advancing speed of the welding tool.

### 3.1. Monte-Carlo Simulation

To generate a set of data points to be used to fit a response surface for the two variable problems we will use a simplified Monte-Carlo simulation method. As there is no statistical uncertainty on the independent variables, we will simply use a uniform distribution to vary the speed of advance and the rotational speed of the tool. A grid of 10 × 10 (*N_p_*= 10, *N_k_* = 10) data points will be used for a total of 100 data points.

**Figure 6.**
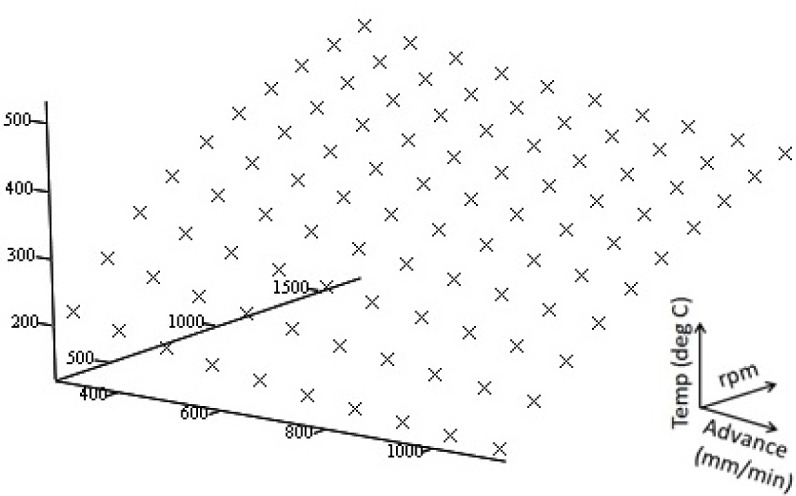
Data points from Monte-Carlo simulation (MCS) results.

The data points (100 points) resulting from the MCS are graphed in Figure 6. The objective function is found to be a polynomial surface, although there are many different methods to create an interpolated function to fit to the obtained data. The linear least squares method is the simplest to implement and gives good results for the function that we are concerned with.

In the MCS, the advancing speed and rotational speed (ω) are uniformly varied according to the following equations:

$$\begin{array}{l} \omega =15.71p+31.4,p=0\ldots N_{p}-1\\ V_{a}=0.0015k+0.005,k=0\ldots N_{k}-1 \end{array}$$ (11)

### 3.2. Least Squares Data Fitting

In this work, we use the least squares method (LSM) to fit a function to the Monte-Carlo data points even though using other methods is certainly possible. A very attractive and elegant method involves re-constructing the data surface with a Bézier surface. Lizheng [17] used a weighted progressive iteration approximation method to iteratively construct Bézier curves to a series of experimentally determined data points.

Park and Kim [18] developed an adaptive method for creating smooth Bézier surfaces for data points using a piecewise cubic triangular Bézier surface possessing C^1^ continuity. Their algorithm effectively reduces the computational time and storage needed of typical smooth surface data surface approximations.

Another interesting method that is gaining increasing popularity is the non-uniform rational B-spline method (NURBS). NURBS surfaces are recognized as being the next logical step in finite element analysis. Yin [19] developed a new algorithm for fitting NURBS surfaces to experimentally determined data points. The method used Lagrange Multipliers to minimize the deviation under boundary constraints.

Polynomial chaos approximations are another interesting analysis method. The method provides a powerful means of evaluating non-Gaussian random variables and stochastic processes. Field and Grigoriu [20] discuss the accuracy of the method, they show that the method suffers from poor approximation for certain types of problems. Soize [21] developed an algorithm to determine high order polynomial chaos expansion with random coefficients. The algorithm is used to solve a problem involving more than a million coefficients through the inverse solution of a stochastic problem. By minimizing the sum of the square of the distance between the fitted coefficients and the data points (Kreyszig [22], Daniel *et al.* [23,24]).

Programming of Bézier curves, NURBS or polynomial chaos adds significant complications for a two parameter optimization model. As such, the linear least squares method will be used. For the LSM surface fitting, the function takes on the general form of:

$$f(x_{1},x_{2})=b_{0}+b_{1}x_{1}{}^{2}+b_{2}x_{1}+c_{1}x_{2}{}^{2}+c_{2}x_{2}+d_{1}x_{1}x_{2}+d_{2}x_{1}{}^{2}x_{2}+d_{3}x_{1}x_{2}{}^{2}$$ (12)

In this case, *x*_1_is the advancing speed and *x*_2_ is the angular speed of the tool. The goal of the LSM is to determine the numeric value of the coefficients (*b_i_*, *c_i_* and *d_i_*). The results of the LSM are:

$$\begin{array}{c} b_{0}=1.588\times 10^{2}\\ b_{1}=9.136\times 10^{-5}\\ b_{2}=-2.187\times 10^{-1}\\ c_{1}=-1.419\times 10^{-4}\\ c_{2}=4.810\times 10^{-1}\\ d_{1}=-7.489\times 10^{-5}\\ d_{2}=-2.497\times 10^{-8}\\ d_{3}=5.506\times 10^{-8} \end{array}$$

The appropriateness of the fit can be evaluated by determining the “*R* squared” value for the fit:

$$R^{2}=1-\left[\frac{\frac{1}{n}\sum_{j=0}^{n-1}{\left(z_{j}-F_{fit}{}_{{}_{j}}\right)}^{2}}{\frac{1}{n}\sum_{j=0}^{n-1}{\left(z_{j}-\mu \right)}^{2}}\right]$$ (13)

where the mean (μ) and standard deviation (σ) are:

$$\mu =\frac{1}{n}\overset{n-1}{\underset{j=0}{\sum }}z_{j}=323.278$$ (14)

$$\sigma =\sqrt{\frac{1}{n}\overset{j=0}{\underset{n-1}{\sum }}{\left(z_{j}-\mu \right)}^{2}}=98.057$$ (15)

**Figure 7.**
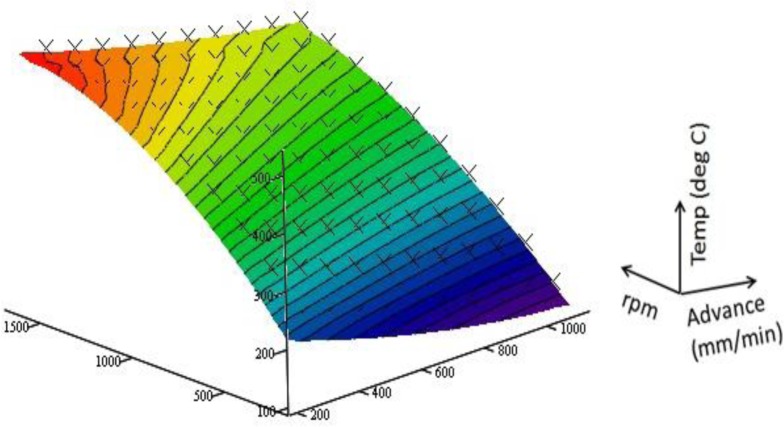
Least square fit graphed with MCS data points.

The *R*^2^ value for the fit is found to be 0.999, thus, the determined function gives a good representation of the MCS data. The resulting surface is graphed against the MCS data points in Figure 7.

### 3.3. Optimization-Exterior Penalty Method

We wish to maximize the speed of advance of the welding tool. The fitted function gives the maximum welding temperature as a function of the welding speed. We need to re-write the fitted function to give the speed of advance as a function of the weld temperature and the rotational speed.

When the fitted function is re-arranged (MathCad root finding routine) to solve for the advancing velocity, there exists two roots to the solutions of the equation. The second root does not give a result within the desired range. As such, the first root is retained for the objective function:

Va(Tweld,ω)=−12(b1+2ωd2)[b2+ω2d3+ωd1+[b2+ω2d1+ω4d32+4Tweldb1−4b0b1+4Tweldωd2−4ωb1c2−4ωb0d2+2ωb2d1−4ω2b1c1+2ω2b2d3−4ω2c2d2−4ω3c1d2+2ω3b1d3]12] (16)

The range of allowable maximum welding temperature, advancing and rotational speeds is taken into consideration by applying constraints to the optimization problem. The constraints are determined from a mixture of economics (welding time should be minimized) and from a concern for the quality of the weld.

From a weld quality standpoint, the maximum temperature at the weld site (*T_weld_*) during the FSW process should be within a certain range. We will use an optimum temperature that was analytically determined by Qian *et al.* [25]:

$$T_{opt}=T_{0}+\left(T_{S}-T_{0}\right)(0.151{\text{log}}_{10}\left(Q^{*}\right)+0.097)$$ (17)

$$Q^{*}=\frac{\sigma_{y@0.8T_{S}}A_{shoulder}C_{p}\eta }{kV_{a}}$$ (18)

$$\eta =\sqrt{\frac{{\left(k\rho C_{p}\right)}_{workpiece}}{{\left(k\rho C_{p}\right)}_{tool}}}$$ (19)

For Al-6061-T6 material, the optimal maximum welding temperature (*T_opt_*) is about 716 K = 443 °C. For the optimization calculation, we will require that the welding temperature be equal to the optimal temperature. The minimum welding speed of advance (*V_a_*) is determined from an economics point of view. In order for the FSW process to compete with common fabrication methods, the speed of advance for the FSW process should not be less than the associated speed of advance for a common steel welded joint. Assuming, for a steel joint, that welding costs ~120 $/h (in a common fabrication shop, that a typical welder is capable of advancing at a rate of 150 mm/min and that the welding cost for a friction stir welded joint is ~160 $/h then an equivalent minimum speed of advance for the FSW process of $150\left(\frac{160}{120}\right)=195\text{mm}/\min$ is required.

The maximum welding speed is due to concerns for the quality of the weld. We will consider for the specific case that we are considering in this work that welding speeds above 1200 mm/min will likely cause welding defects. As such, the constraint on the welding speed is taken to be:

$$195\leq V_{a}\leq 1200$$ (20)

The rotational speed of the tool (ω) is determined based on the typical available rotational speed of a common FSW machine. We will limit the rotational speed to be not less than 200 rpm and not greater than 1800 rpm:

200≤ω≤1800 (21)

### 3.4. Optimization Problem Definition

Maximize:

Va(Tweld,ω)=−12(b1+2ωd2)[b2+ω2d3+ωd1+[b2+ω2d1+ω4d32+4Tweldb1−4b0b1+4Tweldωd2−4ωb1c2−4ωb0d2+2ωb2d1−4ω2b1c1+2ω2b2d3−4ω2c2d2−4ω3c1d2+2ω3b1d3]12] (22)

Subject to:

$$\begin{array}{c} T_{weld}=443\\ g_{1}(T_{weld},\omega )=\omega -1800\leq 0\\ g_{2}(T_{weld},\omega )=200-\omega \leq 0\\ g_{3}(T_{weld},\omega )=195-V_{a}\leq 0\\ g_{4}(T_{weld},\omega )=V_{a}-1200\leq 0 \end{array}$$

The exterior penalty method will be used to solve the optimization problem. This method lends itself well to our problem formulation. Essentially the method will transform the constrained optimization problem into an unconstrained problem by applying a penalty to the objective function. We must write the penalty function based on the objective function and the constraints. Classically the optimization problem is written in order to minimize the objective function. To maximize, we must minimize the negative of the objective function:

$$P(T_{weld},\omega ,\tau_{k})=-V_{a}(T_{weld},\omega )+\tau_{k}\overset{m}{\underset{j=1}{\sum^{\text{​}}}}{\left(\max[g_{j}(x_{1},x_{2}),0]\right)}^{2}$$ (23)

The exterior penalty method algorithm can be summarized by the following steps:

(1) Choose a starting point, $\omega^{\langle 0\rangle }$ that violates one of the constraints;
(2) Use a starting value of τ*_k_* = 1;
(3) Maximize the penalty function to find ω^*^; the solution is stored as $\omega^{\langle k+1\rangle }$;
(4) Check that the calculated value of ω^*^ satisfies the inequality constraint on ω. Also check that the obtained solution satisfies the inequality constraint imposed on the speed of advance, *V_a_*;
(5) If the solution satisfies the constraints, calculate the relative change in the objective function: $\left|\frac{V_{a}(\omega^{\langle k\rangle })-V_{a}(\omega^{\langle k+1\rangle })}{V_{a}(\omega^{\langle k\rangle })}\right|\leq \varepsilon_{tol}$, where ε*_tol_* is the tolerance on the relative change;
(6) If the relative change satisfies the tolerance, the optimal solution is obtained;
(7) If the relative change does not satisfy, increment τ*_k_*_+1_ = 10τ*_k_*;
(8) Repeat steps 3 to 7 until the tolerance on the relative change is satisfied.

In order to solve the problem, we start by choosing an initial point to begin the optimization algorithm. We will choose a value of ω that violates the g_1_ constraint:

$$\omega^{\langle 0\rangle }=1805$$ (24)

**Figure 8.**
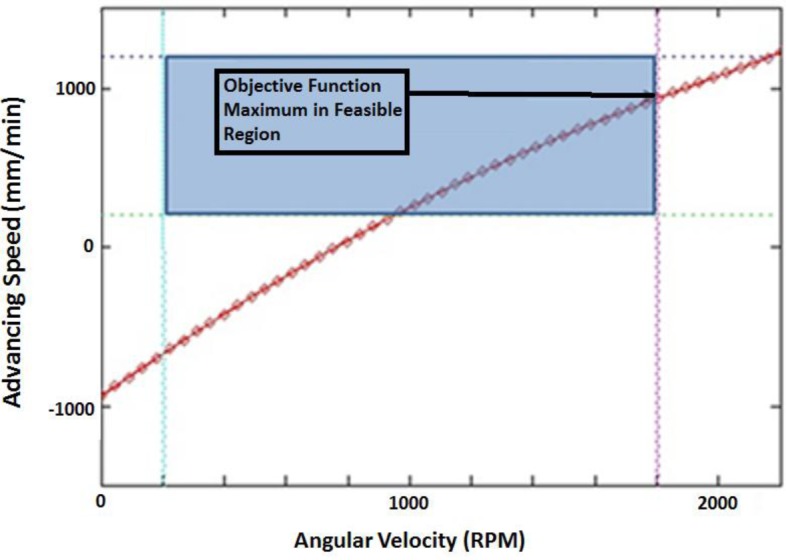
Feasible region.

Thus, we will be approaching the feasible region (shown in Figure 8) from the outside of the g_1_ constraint. Now we will minimize the penalty function by taking the derivative with respect to ω and setting it to zero:

$$\frac{\partial }{\partial \omega }P\left(\omega ,\tau_{k}\right)=0$$ (25)

**Figure 9.**
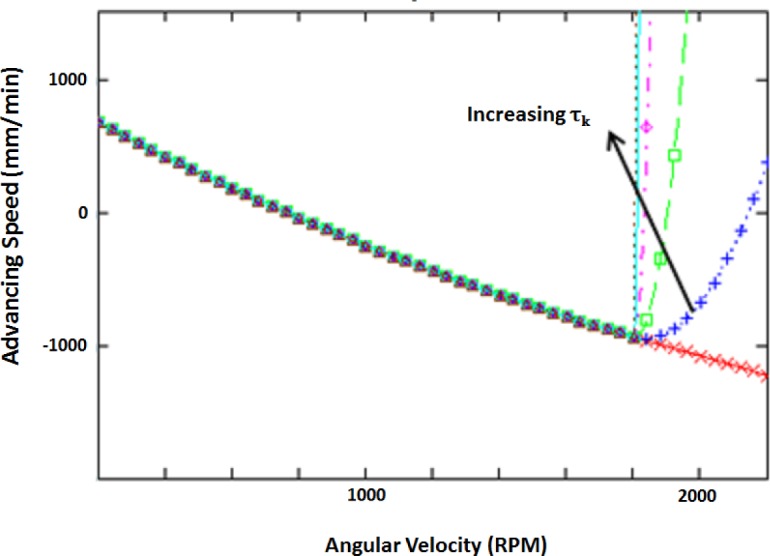
Graphical representation of penalty functions.

Solving for the roots of the resulting equation is not a trivial task. An iterative approach is used to find the optimum value of ω. The results of the optimization are shown in Table 2. A graph showing the penalty function for various values of τ*_k_* is shown in Figure 9.

Notice that the penalty function passes through the optimum for all values of τ*_k_*, this is because the optimal value is on the boundary of the feasible region and because the function is monotonically decreasing.

## 4. Results of the FDM Simulation at Optimal Values

**Figure 10.**
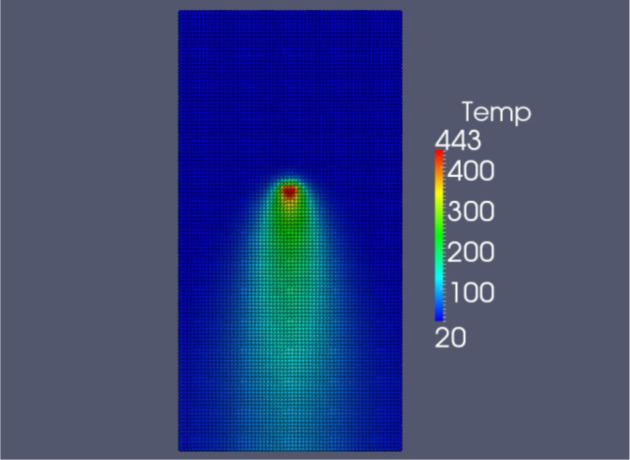
Temperature results from FDM simulation at optimal parameters, *t* = 23 s.

**Figure 11.**
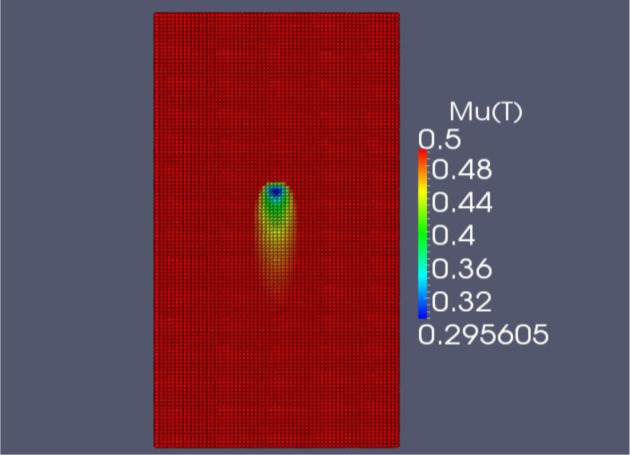
μ*_k_*(*T*) results from FDM simulation at optimal parameters, *t* = 23 s.

The FDM code is programmed in C++, the explicit time integration allows for a straight forward marching algorithm. The results of the FDM simulation of the friction stir welding process at the optimal parameters (*V_a_* = 934.3 mm/min and ω = 1800 rpm) are shown in Figure 10 when *t* = 23 s. Note that the maximum weld temperature is 443 °C. Paraview 3.12.0 (for windows) is used to visualize the results. The coefficient of friction is shown in Figure 11. We can see that μ*_k_* is at a minimum value directly under the tool.

### Discussion of Results

The proposed optimization method in this work provides a means for determining the optimum parameters of the process either numerically or experimentally. The algorithm would only require slight modification to be used for experimental data.

The choice of the temperature dependence of the coefficient of friction has an important effect on the optimization results. In this work, we choose a friction dependence that involves a decrease in the coefficient of friction as the temperature increases. Had another friction dependence been defined, a completely different optimal tool speed would have been found.

The temperature distribution in the aluminum work pieces does not vary through the thickness of the plate. This is because the FDM model was developed in two dimensions. Certainly, during the actual FSW process, the temperature varies through the thickness of the plate. In this work, we are mainly concerned with the maximum welding temperature. As such, the 2D model is a very reasonable approximation.

## 5. Comparison with Experiments

Experimental optimization of the friction stir welding process has been carried out by many groups. We will compare the results of our numerical optimization technique to those obtained by Wanjara *et al.* [26]. In their work, they perform experimental optimization of 6061-T6 butt welded plates that are 3.18 mm thick. They find that there exists an optimal window of operation for the FSW process.

In our numerical optimization procedure, all the FSW parameter ranges, plate thickness and tool dimensions are the same as those taken by Wanjara *et al.* [26]. They note that a weld pitch of ~0.48 mm/rev is ideal for their setup.

**Figure 12.**
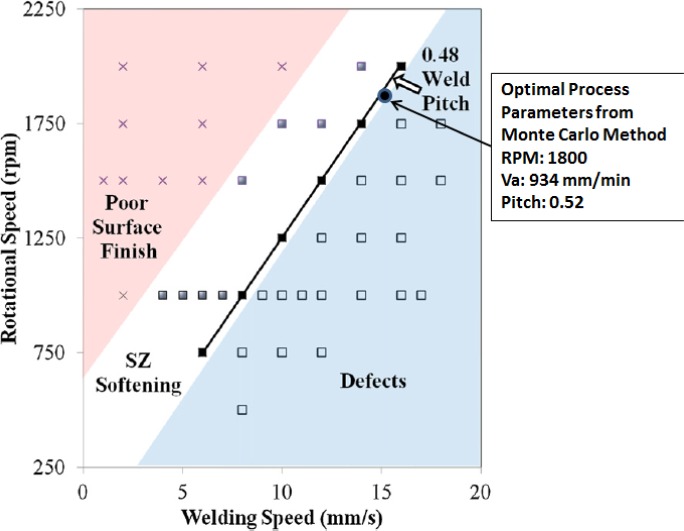
Processing map for FSW of 3.18mm 6061-T6 plate [26] (Adapted from [26]).

The optimal process parameters that we have found from the numerical optimization approach for the 3.18 mm 6061-T6 plate is 1800 rpm with an advance speed of 934 mm/min. This gives a weld pitch of 0.52, this is in very good agreement with the experimental findings of Wanjara *et al.* [26]. The optimal parameters are on the edge of the operational window (see Figure 12) since the goal of the optimization is to obtain the greatest possible speed of advance without the occurrence of defects.

## 6. Conclusions and Future Work

The exterior penalty optimization method was used to determine the optimum welding speed of advance and angular velocity of the tool. The optimum values are found to be:

$$\begin{array}{c} T_{weld}=443\;C\\ V_{a}=934\frac{\text{mm}}{\min}\\ \omega =1800\;\text{rpm} \end{array}$$

The following conclusions can be drawn from this work:

- The temperature distribution during the FSW process can be determined using a non-linear finite difference formulation;
- The Monte-Carlo simulation method is a powerful tool that can be used for optimization of the FSW process;
- The exterior penalty optimization method lends itself well to this simplified two parameter model;
- Extension to a higher number of parameters is possible with the exterior penalty method;
- Although the simplified numerical model only takes into account the evolution of the temperature field during the FSW process, the microstructure change in the joint is very closely related to the temperature field;
- A estimate of the optimal FSW process parameters can be obtained from a simplified numerical model that approximates the joint strength and microstructure using the temperature field;
- The proposed optimization approach is in close agreement with experimental optimization work;
- Using this technique, optimal process parameters can be found in less than 30 min of calculation time.

Future work will involve creating a built-in optimization method within the FDM code (or a sub-program designed to interact with a commercial finite element code like LS-DYNA). The user would decide on a starting point, and the code would iteratively progress through the calculations by choosing new parameters and testing for optimality. The sequential simplex method [27] could be used for such a procedure. The idea is for the code to calculate the temperature distribution in the plates for three sets of initial parameters. Then the algorithm would reject the worst set of the three, and would choose a new parameter set based on the previous three sets. The solution would progress iteratively until the desired convergence tolerance is obtained.

## Figures and Tables

**Table 1.** Simulation parameters.

Parameter	Value	Units
*k_cond_*	175	W/mK
*C_p_*	875	J/kg·Kelvin
ρ	2700	kg/m^3^
Tool diameter	0.013	m
Pin diameter	0.005	m
Plate length	0.5	m
Plate width	0.25	m
*t_plate_*	0.0032	m
Initial temperature	20	°C
*T*_∞_	20	°C
$\bar{h}$	15	W/m^2^·K
*R″_tc_*	2.75	m^2^·K/W
*k_steel_*	60	W/m·K
*t_steel_*	0.05	m

**Table 2.** Optimization results.

Parameter	Value	Units
Rotational speed (ω)	1800	rpm
Temperature	443	°C
Advancing speed (*V_a_*)	934.3	mm/min
